# Non-contiguous finished genome sequence and description of *Bacteroides neonati* sp. nov., a new species of anaerobic bacterium

**DOI:** 10.4056/sigs.5159098

**Published:** 2014-04-04

**Authors:** Nadim Cassir, Olivier Croce, Isabelle Pagnier, Samia Benamar, Carine Couderc, Catherine Robert, Didier Raoult, Bernard La Scola

**Affiliations:** 1 Unité de Recherche sur les Maladies Infectieuses et Tropicales Emergentes (URMITE), Facultés de Médecine et de Pharmacie, Aix-Marseille Université, France.

**Keywords:** *Bacteroides neonati*, genome

## Abstract

*Bacteroides neonati* strain MS4^T^, is the type strain of *Bacteroides neonati* sp. nov., a new species within the genus *Bacteroides*. This strain, whose genome is described here, was isolated from a premature neonate stool sample. *B. neonati* strain MS4^T^ is an obligate anaerobic Gram-negative bacillus. Here we describe the features of this organism, together with the complete genome sequence and annotation. The 5.03 Mbp long genome exhibits a G+C content of 43.53% and contains 4,415 protein-coding and 91 RNA genes, including 9 rRNA genes.

## Introduction

*Bacteroides neonati* strain MS4^T^ (= CSUR P 1500= DSM 26805), is the type strain of *Bacteroides neonati* sp. nov., and a new member of the genus *Bacteroides*. This bacterium is a Gram-negative, anaerobic, non spore-forming, indole positive bacillus that was isolated from a preterm neonate stool sample, during a study prospecting stool samples from patients with necrotizing enterocolitis and controls [unpublished].

To define a new bacterial species or genus, the “gold standard” method is the DNA-DNA hybridization and G+C content determination [1]. However, those methods are expensive, and poorly reproducible. The development of PCR and sequencing methods led to new ways of classifying bacterial species, using in particular 16S rDNA sequences with an internationally-validated cutoff value [2]. More recently, new bacterial genera and species are described using high throughput genome sequencing and mass spectrometric analyses, which allow access to a wealth of genetic and proteomic information [3,4]. We propose the description of a new bacterial species, using genome sequences, MALDI-TOF spectra, and the main phenotypic characteristics, as previously done [5-22].

Here we present a summary classification and a set of features for *B. neonati* sp. nov. strain MS4^T^ (= CSUR P 1500= DSM 26805) together with a description of the complete genomic sequencing and annotation. These characteristics support the circumscription of a novel species, *B. neonati* sp. nov., within the *Bacteroides* genus.

The *Bacteroidaceae* family is currently comprised of 3 genera: *Acetomicrobium*, *Anaerorhabdus* and *Bacteroides*. It is a heterogeneous family, grouping anaerobic and morphologically variable bacteria, and it is defined mainly on the basis of phylogenetic analyses of 16S rDNA sequences. The most closely related species to *Bacteroides neonati* sp. nov. is *Bacteroides graminisolvens* [23] followed by *Bacteroides intestinalis* [24]. *Bacteroides neonati* is a strictly anaerobic Gram negative, non spore-forming bacterium.

## Classification and features

**Figure 1:**
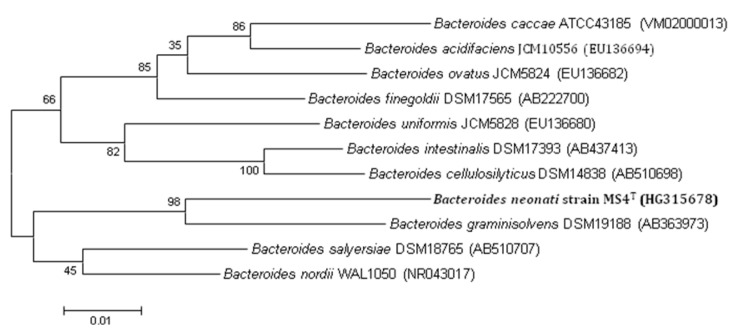
Phylogenetic tree highlighting the position of *Bacteroides neonati* MS4^T^ relative to other type strains within the genus *Bacteroides*. GenBank accession numbers are indicated in parentheses. Sequences were aligned using CLUSTALW, and phylogenetic inferences obtained using the maximum-likelihood method within the MEGA 4 software [39]. Numbers at the nodes are bootstrap values obtained by repeating the analysis 500 times the analysis to generate a majority consensus tree. The scale bar represents a 2% nucleotide sequence divergence.

A stool sample was collected from a patient during a case-control study analyzing the fecal microbiota of premature neonates with necrotizing enterocolitis, using MALDI-TOF and 16S rRNA gene sequencing [unpublished]. After collection in Marseille, the specimen was preserved at -80°C. Strain MS4^T^ (Table 1) was isolated in October 2012, by anaerobic cultivation on 5% sheep blood-enriched Columbia agar (BioMerieux, Marcy l’Etoile, France). This strain exhibited a 94% nucleotide sequence similarity with *Bacteroides graminisolvens* [23] and a 94% nucleotide sequence similarity with *Bacteroides intestinalis* [24]. Those similarity values are lower than the threshold recommended to delineate a new species without carrying out DNA-DNA hybridization [38]. In the inferred phylogenetic tree, it forms a distinct lineage close to *Bacteroides graminisolvens* (Figure 1).

**Table 1:** Classification and general features of *Bacteroides neonati* strain MS4^T^ according to the MIGS recommendations [25]

**MIGS ID**	**Property**	**Term**	**Evidence code^a^**
		Domain *Bacteria*	TAS [26]
		Phylum *Bacteroidetes*	TAS [27,28]
		Class *Bacteroidia*	TAS [27,29]
	Current classification	Order *Bacteroidales*	TAS [27,30]
		Family *Bacteroidaceae*	TAS [31,32]
		Genus *Bacteroides*	TAS [31,33-36]
		Species *Bacteroides neonati*	IDA
		Type strain MS4	IDA
	Gram stain	Negative	IDA
	Cell shape	Bacillus	IDA
	Motility	Non motile	IDA
	Sporulation	Non spore-forming	IDA
	Temperature range	Mesophile	IDA
	Optimum temperature	37°C	IDA
MIGS-6.3	Salinity	Weak growth on BHI medium + 1% NaCl	IDA
MIGS-22	Oxygen requirement	Anaerobic	IDA
	Carbon source	Unknown	NAS
	Energy source	Unknown	NAS
MIGS-6	Habitat	Gut	IDA
MIGS-15	Biotic relationship	Free living	IDA
MIGS-14	Pathogenicity Biosafety level Isolation	Unknown 2 Stool sample	NAS
MIGS-4	Geographic location	France	IDA
MIGS-5	Sample collection time	October 2012	IDA
MIGS-4.1	Latitude	43.296482	IDA
MIGS-4.1	Longitude	5.36978	IDA
MIGS-4.3	Depth	Surface	IDA
MIGS-4.4	Altitude	0 above see level	IDA

**^a^**Evidence codes - IDA: Inferred from Direct Assay; TAS: Traceable Author Statement (i.e., a direct report exists in the literature); NAS: Non-traceable Author Statement (i.e., not directly observed for the living, isolated sample, but based on a generally accepted property for the species, or anecdotal evidence). These evidence codes are from the Gene Ontology project [37]. If the evidence is IDA, then the property was directly observed for a live isolate by one of the authors or an expert mentioned in the acknowledgments.

Seven different growth temperatures (23°C, 25°C, 28°C, 32°C, 35°C, 37°C, 50°C) were tested; no growth occurred at 50°C, growth occurred between 23° and 37°C, and optimal growth was observed at 37°C.

**Figure 2:**
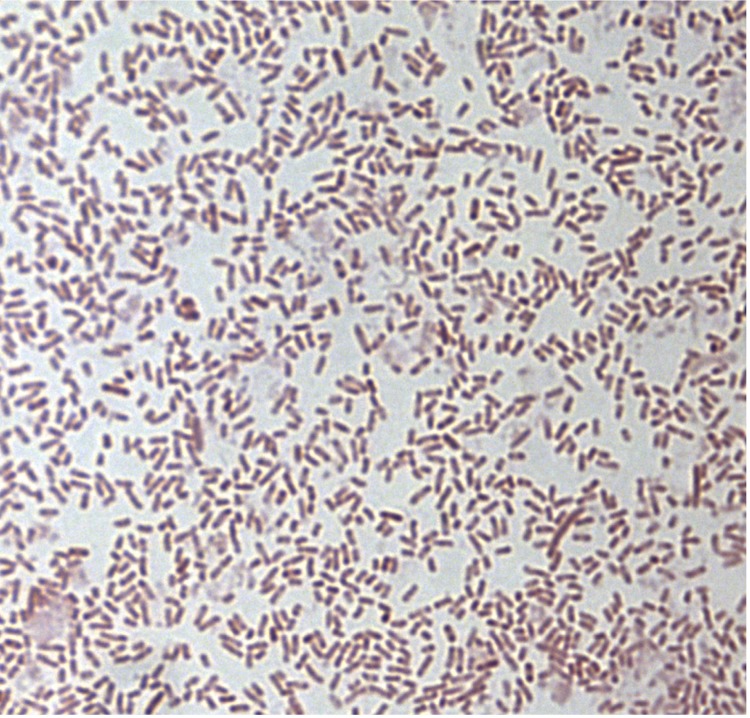
Gram staining of *B. neonati* strain MS4^T^

**Figure 3:**
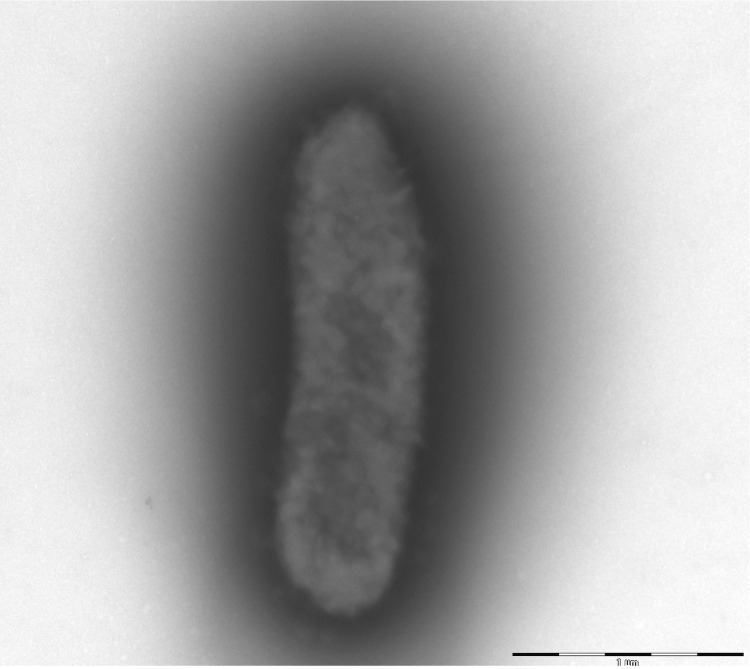
Transmission electron micrograph of *B. neonati* strain MS4^T^, using a Morgani 268D (Philips) at an operating voltage of 60kV. The scale bar represents 1,000 nm.

Colonies are punctiform, medium-sized, grey, shiny and round on blood-enriched Columbia agar under anaerobic conditions using GENbag anaer (BioMérieux). Bacteria were grown on blood-enriched Columbia agar (Biomerieux) and in Trypticase-soy TS broth medium, under anaerobic conditions using GENbag anaer (BioMérieux). They also were grown under anaerobic conditions on BHI agar and on BHI agar supplemented with 1% NaCl. Growth was achieved only anaerobically on blood-enriched Columbia agar and weakly on BHI agar as well as BHI agar supplemented with 1% NaCl after 72h incubation. Gram staining showed plump non spore-forming Gram-negative bacilli (Figure 2). The motility test was negative. Cells grow anaerobically in TS broth medium have a mean wide of 0.681 µm (min = 0.323 µm; max = 0.878 µm) and a mean length of 2.165 µm (min = 1.402; max = 2.951), as determined using electron microscopic observation after negative staining (Figure 3).

Strain MS4^T^ exhibited catalase activity but no oxidase activities. Using API 20A, a positive reaction could be observed only weekly for Gelatinase. Using Api Zym, a positive reaction was observed for alkaline phosphatase (40 nmol of hydrolyzed substrata), acid phosphatase (40 nmol), naphtolphosphohydrolase (20 nmol), esterase (20 nmol), esterase lipase (5 nmol), alpha-galactosidase (5 nmol), beta-galactosidase (20 nmol), beta-glucuronidase (30 nmol), beta-glucosidase (5 nmol), N-acetyl-beta-glucosaminidase (40 nmol) and alpha-fucosidase (5 nmol). Using Api rapid id 32A, a positive reaction was observed for alpha-galactosidase, alpha-glucosidase, N-acetyl-beta-glucosaminidase and alpha-fucosidase. Regarding antibiotic susceptibility, *Bacteroides neonati* was susceptible to clavulanate-amoxicillin, imipenem and metronidazole. When compared to the representative species within the genus *Bacteroides*, *B. neonati* exhibits the phenotypic characteristics detailed in Table 2 [40].

**Table2:** Differential characteristics of *Bacteroides neonati* sp. nov., strain MS4^T^, *B. graminisolvens* strain DSM 19988^T^, and *B. intestinalis* strain DSM 17397^T^.

**Properties**	*B. neonati*	*B. graminisolvens*	*B. intestinalis*
Cell wide (µm) Cell long (µm)	0.3–0.8 1.4–2.9	0.4–0.6 1.2–4.5	n.a. 1–3
Oxygen requirement	Anaerobic	Anaerobic	Anaerobic
Gram stain	Negative	Negative	Negative
Optimal growth temperature	37°C	35°C	37°C
Habitat	Human	Methanogenic reactor	Human
			
**Enzyme production**			
Indole	-	-	+
Alkaline Phosphatase	+	-	+
Urease	-	-	-
Catalase	+	-	n.a.
Gelatinase	+	-	-
			
**Utilization of**			
Glucose	-	+	+
Mannose	-	+	+
Lactose	-	+	+
Raffinose	-	+	+

**Figure 4:**
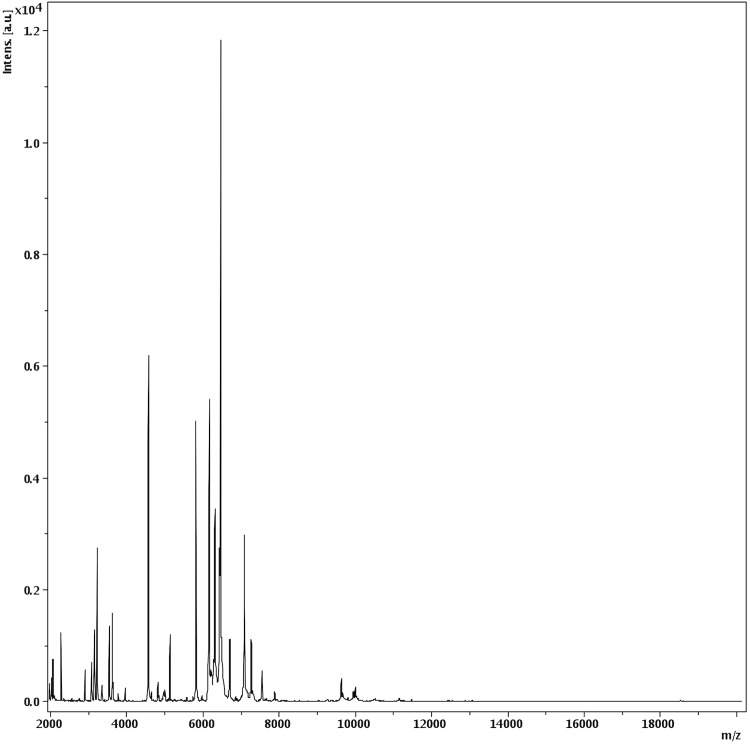
Reference mass spectrum from *B. neonati* strain MS4^T^. Spectra from 10 individual colonies were compared and a reference spectrum was generated.

**Figure 5:**
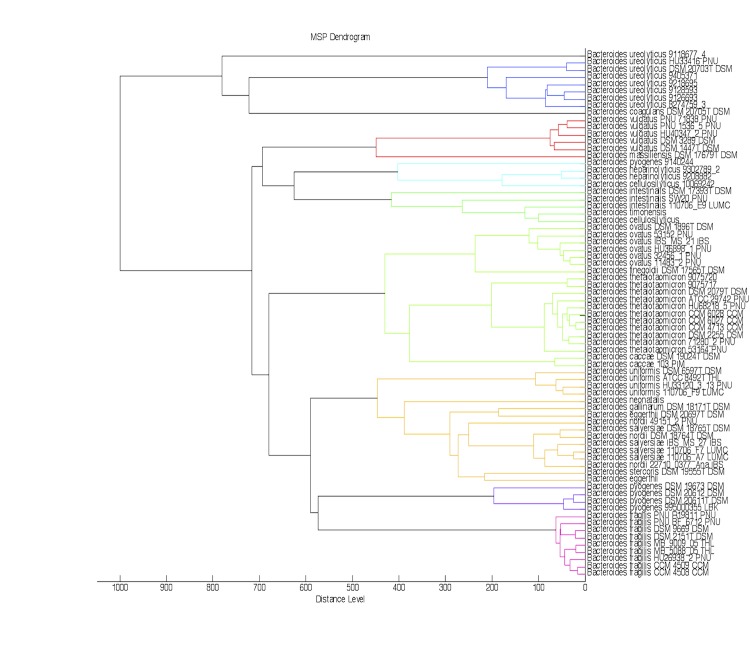
Dendrogram based on the comparison of the *B. neonati* strain MS4^T^ MALDI-TOF reference spectrum, and 72 other species of the genus of *Bacteroides.*

Matrix-assisted laser-desorption/ionization time-of-flight (MALDI-TOF) MS protein analysis was carried out as previously described [41]. A pipette tip was used to pick one isolated bacterial colony from a culture agar plate, and to spread it as a thin film on a MTP 384 MALDI-TOF target plate (Bruker Daltonics, Germany). Ten distinct deposits were done for strain MS4^T^ from ten isolated colonies. Each smear was overlaid with 2 µL of matrix solution (saturated solution of alpha-cyano-4-hydroxycinnamic acid) in 50% acetonitrile, 2.5% tri-fluoracetic acid, and allowed to dry for five minutes. Measurements were performed with a Microflex spectrometer (Bruker). Spectra were recorded in the positive linear mode for the mass range of 2,000 to 20,000 Da (parameter settings: ion source 1 (ISI), 20kV; IS2, 18.5 kV; lens, 7 kV). A spectrum was obtained after 675 shots at a variable laser power. The time of acquisition was between 30 seconds and 1 minute per spot. The ten MS4^T^ spectra were imported into the MALDI Bio Typer software (version 2.0, Bruker) and analyzed by standard pattern matching (with default parameter settings) against the main spectra of 6,335 bacteria, in the Bio Typer database. The method of identification includes the m/z from 3,000 to 15,000 Da. For every spectrum, 100 peaks at most were taken into account and compared with the spectra in database. A score enabled the identification, or not, from the tested species: a score > 2 with a validated species enabled the identification at the species level; a score > 1.7 but < 2 enabled the identification at the genus level; and a score < 1.7 did not enable any identification. For strain MS4^T^, the best-obtained score was 1.345, which is not significant, suggesting that our isolate was not a member of a known genus. The reference spectrum from strain MS4^T^ (Figure 4) was added to our database. A dendrogram was constructed with the MALDI Bio Typer software (version 2.0, Bruker), comparing the reference spectrum of strain MS4 with reference spectra of 26 bacterial species, all belonging to the order of *Bacteroidetes*. In this dendrogram, strain MS4^T^ appears as a separated branch within the genus *Bacteroides* (Figure 5).

## Genome sequencing and annotation

### Genome project history

The organism was selected for sequencing because it was isolated from a premature neonate stool sample as part of a study prospecting stool samples from patients with necrotizing enterocolitis.

The Genbank accession number is HG726019 - HG726036 and consists of 18 scaffolds with a total of 35 contigs. Table 3 shows the project information and its compliance with MIGS version 2.0 standards.

**Table 3:** Project information

**MIGS ID**	**Property**	**Term**
MIGS-31	Finishing quality	Non-contiguous finished
MIGS-28	Libraries used	One 454 PE 3-kb library
MIGS-29	Sequencing platforms	454 GS FLX+ Titanium
MIGS-31.2	Sequencing coverage	27.0
MIGS-30	Assemblers	Newbler 2.8
MIGS-32	Gene calling method	Prodigal 2.5
	Genbank ID	HG726019 - HG726036
	Genbank Date of Release	November, 2013
MIGS-13	Source material identifier	DSM 26805
	Project relevance	Stool samples from patients with necrotizing enterocolitis

### Growth conditions and DNA isolation

*Bacteroides neonati* strain MS4^T^ (= CSUR P 1500= DSM 26805), was grown on blood agar medium at 37°C under anaerobic conditions. Eight petri dishes were spread and resuspended in 5 ×100µl of G2 buffer. A first mechanical lysis was performed using glass powder in the Fastprep-24 Sample Preparation system (MP Biomedicals, USA) with 2×20 second bursts. DNA was then incubated with lysozyme (30 minutes at 37°C) and extracted on a BioRobot EZ 1 Advanced XL (Qiagen). The DNA was then concentrated and purified on a Qiamp kit (Qiagen). The yield and the concentration were measured by the Quant-it Picogreen kit (Invitrogen) on the Genios_Tecan fluorometer at 15.7ng/µl.

### Genome sequencing and assembly

A 3 kb paired end library was pyrosequenced on the 454 Roche Titanium. This project was loaded on a 1/4 region on PTP Picotiterplates. 5 µg of DNA was mechanically fragmented with a Hydroshear device (Digilab, Holliston, MA, USA) with an enrichment size at 3-4kb. The DNA fragmentation was visualized with an Agilent 2100 BioAnalyzer on a DNA labchip 7500 with an average size of 3.2 kb. The library was constructed according to the 454 Titanium paired end protocol supplied by the manufacturer. Circularization and nebulization were performed and generated a pattern with an optimal at 604 bp. After PCR amplification through 15 cycles followed by double size selection, the single stranded paired end library was then quantified on the Agilent 2100 BioAnalyzer on a RNA pico 6,000 labchip at 91pg/µL. The library concentration equivalence was calculated at 2.76 x 10^8^ molecules/µL. The library was stored at -20°C until used.

The library was clonally amplified with 0.5 and 1 cpb in 2 emPCR reactions in each condition with the GS Titanium SV emPCR Kit (Lib-L) v2. The yield of the emPCR was 10.46 and 11.53%, respectively, according to the quality expected by the range of 5 to 20% from the Roche procedure. 790,000 beads were loaded on the GS Titanium PicoTiterPlates PTP Kit 70×75 sequenced with the GS Titanium Sequencing Kit XLR70

The 454 sequencing generated 811,269 reads (180 Mb, coverage of 27.0) assembled into contigs and scaffolds using Newbler version 2.8 (Roche, 454 Life Sciences) and Mira assembler v3.2 [42]. The obtained contigs were combined using the Opera software v1.2 [43] in tandem with GapFiller V1.10 [44] to reduce the set. Finally, some manual refinements using CLC Genomics software v4.7.2 (CLC bio, Aarhus, Denmark) were made. The genome consists of 35 contigs in18 scaffolds.

### Genome annotation

Non-coding genes and miscellaneous features were predicted using RNAmmer [45], ARAGORN [46], Rfam [47], PFAM [48]. Open Reading Frames (ORFs) were predicted using Prodigal [49] with default parameters but the predicted ORFs were excluded if they spanned a sequencing GAP region. The functional annotation was achieved using BLASTP [50] against the GenBank database [51] and the Clusters of Orthologous Groups (COGs) database [52,53].

## Genome properties

**Figure 6:**
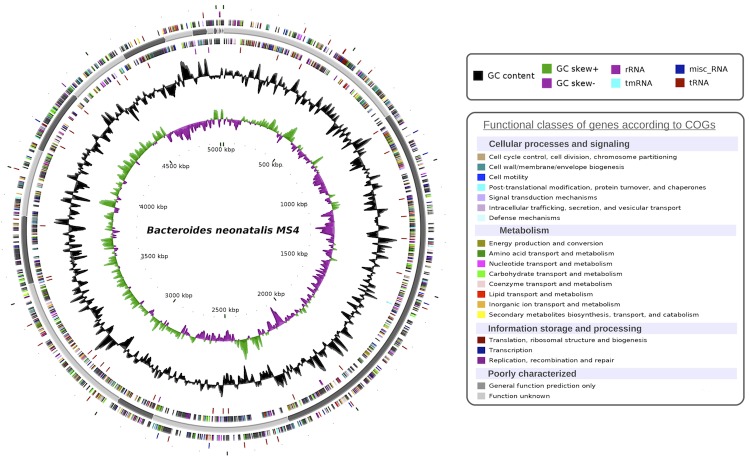
Circular representation of the *Bacteroides neonati* chromosome. Circles from the center to the outside: GC skew (green/purple), GC content (black), tRNA (dark red), rRNA (purple), tmRNA (blue), miscellaneous RNA (deep blue) on forward strand, genes on forward strand colored by COGs categories, scaffolds in alternative grays, genes on reverse strand colored by COGs, tRNA (dark red), rRNA (purple), tmRNA (blue), miscellaneous RNA (deep blue) on reverse strand.

The genome of *B. neonati* strain MS4^T^ is estimated to be 5.03 Mb long with a G+C content of 43.53% (Figure 6 and Table 4). A total of 4,415 protein-coding and 91 RNA genes, including 9 rRNA genes, 65 tRNA, 1 tmRNA and 39 miscellaneous other RNA were founded. The majority of the protein-coding genes were assigned a putative function (69.26%) while the remaining ones were annotated as hypothetical proteins. The properties and the statistics of the genome are summarized in Table 4. The distribution of genes into COG functional categories is presented in Table 5.

**Table 4:** Nucleotide content and gene count levels of the genome

**Attribute**	**Value**	% of Total*
Genome size (bp)	5,026,786	100
DNA coding region (bp)	4,556,154	90.63
DNA G+C content (bp)	2,188,298	43.53
Total genes	4506	100
rRNA	9	0.18
tRNA	65	0.1
tmRNA	1	0.01
miscRNA	39	0.05
Protein-coding genes	4415	97.98
Genes with function prediction	3121	69.26
Genes assigned to COGs	4303	97.46

* The total is based on either the size of the genome in base pairs or the total number of protein coding genes in the annotated genome.

**Table 5:** Number of genes associated with the 25 general COG functional categories.

**Code**	**Value**	% age*	**Description**
J	225	4.73	Translation
A	15	0.31	RNA processing and modification
K	280	5.89	Transcription
L	313	6.58	Replication, recombination and repair
B	10	0.21	Chromatin structure and dynamics
D	84	1.77	Cell cycle control, mitosis and meiosis
Y	3	0.06	Nuclear structure
V	90	1.89	Defense mechanisms
T	189	3.97	Signal transduction mechanisms
M	317	6.66	Cell wall/membrane biogenesis
N	35	0.73	Cell motility
Z	11	0.23	Cytoskeleton
W	0	0	Extracellular structures
U	135	2.84	Intracellular trafficking and secretion
O	187	3.93	Posttranslational modification, protein turnover, chaperones
C	271	5.69	Energy production and conversion
G	309	6.49	Carbohydrate transport and metabolism
E	300	6.31	Amino acid transport and metabolism
F	105	2.21	Nucleotide transport and metabolism
H	200	4.2	Coenzyme transport and metabolism
I	112	2.35	Lipid transport and metabolism
P	336	7.06	Inorganic ion transport and metabolism
Q	66	1.39	Secondary metabolites biosynthesis, transport and catabolism
R	568	11.93	General function prediction only
S	486	10.21	Function unknown
-	112	2.35	Not in COGs

* The total is based on the total number of protein coding genes in the annotated genome.

## Insights into the genome sequence

We made some brief comparisons against *Bacteroides intestinalis* DSM 17393 (ABJL00000000) that is currently the closest available sequenced genome. This genome is composed of 8 contigs (ABJL02000001-ABJL02000008).

The draft genome sequence of *Bacteroides neonati* has a smaller size compared to the *Bacteroides intestinalis* (respectively, 5.03 Mb against 6.05 Mb). The G+C content is very close to *Bacteroides intestinalis* (respectively, 43.53% and 42.8%). *Bacteroides neonati* has slightly fewer genes (4,506 genes against 4,984 genes), and a higher ratio of genes per Mb (895.82 genes/Mb against 823.8 genes/Mb).

Table 6 presents the difference of gene number (in percentage) related to each COG category between *Bacteroides neonati* and *Bacteroides intestinalis*. The proportion of COG is highly similar between the two species. The maximum difference is related to the COG "Carbohydrate Metabolism and transportation" which does not exceed 2.28%.

**Table 6:** Percentage of genes associated with the 25 general COG functional categories for *B. neonati* and *B. intestinalis* DSM 20548.

**Code**	***B. neonati***** **%age**	***B. intestinalis*** **%age**	**% Difference**	**COG description**
J	4.73	4.26	0.47	Translation, ribosomal structure and biogenesis
A	0.31	0.24	0.07	RNA processing and modification
K	5.89	5.63	0.26	Transcription
L	6.58	6.21	0.37	Replication, recombination and repair
B	0.21	0.21	0	Chromatin structure and dynamics
D	1.77	1.49	0.28	Cell cycle control, cell division, chromosome partitioning
Y	0.06	0.02	0.04	Nuclear structure
V	1.89	2.46	-0.57	Defense mechanisms
T	3.97	4.68	-0.71	Signal transduction mechanisms
M	6.66	7.46	-0.8	Cell wall/membrane biogenesis
N	0.73	1.11	-0.38	Cell motility
Z	0.23	0.12	0.11	Cytoskeleton
W	0	0.02	-0.02	Extracellular structures
U	2.84	2.94	-0.1	Intracellular trafficking and secretion, and vesicular transport
O	3.93	3.45	0.48	Posttranslational modification. protein turnover, chaperones
C	5.69	4.58	1.11	Energy production and conversion
G	6.49	8.77	-2.28	Carbohydrate transport and metabolism
E	6.31	5.53	0.78	Amino acid transport and metabolism
F	2.21	1.97	0.24	Nucleotide transport and metabolism
H	4.2	4.09	0.11	Coenzyme transport and metabolism
I	2.35	2.2	0.15	Lipid transport and metabolism
P	7.06	6.21	0.85	Inorganic ion transport and metabolism
Q	1.39	1.37	0.02	Secondary metabolites biosynthesis, transport and catabolism
R	11.93	12.62	-0.69	General function prediction only
S	10.21	10	0.21	Function unknown
-	2.35	2.33	0.02	Not in COGs

## Conclusion

On the basis of phenotypic, phylogenetic and genomic analysis, we formally propose the creation of *Bacteroides neonati* that contains the strain MS4^T^. This bacterium has been found in Marseille, France.

### Description of *Bacteroides neonati* sp. nov.

Bacteroides neonati (neo.na’ti L. gen. masc. n. neonati, because this new species has been first isolated from a preterm neonate stool sample)is a Gram-negative bacillus; Obligate anaerobic; Non-spore-forming bacterium; Grows on axenic medium at 37°C in anaerobic atmosphere; Negative for indole; Non motile; The G+C content of the genome is 43.53%. The type strain is MS4^T^ (= CSUR P 1500 = DSM 26805).
